# Temporal trends and future projections of six major cancers among women of childbearing age in northeast Asia: analysis of data from the global burden of disease study 2021

**DOI:** 10.3389/fonc.2025.1716310

**Published:** 2025-11-28

**Authors:** Jingqian Liang, Baogeng Huai, Zeping Yang, Shan Zhang, Haoliang Cui, Jianyi Zhang, Deshan Liu

**Affiliations:** 1 Jinan Zhangqiu District Hospital of Traditional Chinese Medicine, Jinan, China; 2 Department of Traditional Chinese Medicine, Qilu Hospital of Shandong University, Jinan, Shandong, China; 3 Vanke School of Public Health, Tsinghua University, Beijing, China; 4 School of Public Health, Peking University, Beijing, China

**Keywords:** Global Burden of Disease study, Women of child-bearing age, Northeast Asia, breast cancer, cervical cancer, uterine cancer, ovarian cancer, colon and rectum cancer

## Abstract

**Background:**

Cancer among women of childbearing age (WCBA) imposes major health and socioeconomic burdens, yet integrated assessments of temporal trends and future projections in Northeast Asia (NEA) are limited.

**Methods:**

Using Global Burden of Disease 2021 estimates, we quantified incidence, mortality, and disability-adjusted life years (DALYs) for breast, cervical, uterine, ovarian, colorectal (CRC), and tracheal, bronchus, and lung (TBL) cancers among women of childbearing age (WCBA; 15–49 years) in six Northeast Asia countries from 1990–2021. We calculated age-standardized incidence, mortality and DALY rates, temporal odds ratios for the proportion of WCBA among all female cases, estimated annual percentage changes (EAPCs), and mortality-to-incidence ratios (MIRs), referencing a 46.6% threshold. Age-standardized incidence and mortality rates to 2050 were projected using Bayesian age–period–cohort models fitted with integrated nested Laplace approximation.

**Findings:**

Between 1990 and 2021, the proportion of cancer incidence, deaths, and DALYs among WCBA relative to all females declined across all cancer types. The steepest proportional declines were for TBL incidence ( $OR_{trend}$ = 0.972, 95% CI: 0.969 to 0.976) and CRC deaths ( $OR_{trend}$ = 0.967, 95% CI: 0.965–0.969). MIRs remained below the 46.6% threshold for breast and cervical cancers, while TBL cancers persistently exceeded it (β = –0.006, 95% CI: –0.007 to –0.006). From 1990–2021, ASIRs increased for breast, cervical (EAPC = 1.58%, 95% CI: 1.49 to 1.66), cervical, and uterine cancers but decreased for ovarian cancer decreased (EAPC = –0.44%, 95% CI: –0.53 to –0.36). ASMRs declined for all cancers, most sharply for uterine cancer (EAPC = –2.38%, 95% CI: –2.68 to –2.09). ASDRs declined across cancers, most prominently for CRC (EAPC = –2.30%, 95% CI: –2.49 to –2.12). Projections to 2050 indicate continued increases in breast and uterine cancer incidence, stabilization of CRC and TBL incidence, and a sustained high cervical cancer burden in Mongolia and DPRK. Breast, uterine, and TBL cancer mortality are projected to remain stable.

**Interpretation:**

Although mortality and DALYs among WCBA in NEA have improved, rising breast and uterine cancer incidence, persistently high lung cancer MIRs remain major concerns. Strengthened tobacco control and air-quality policies, expanded HPV vaccination and cervical screening, promotion of healthy lifestyles, and equitable access to early diagnosis and treatment are essential to reduce cross-country disparities in cancer outcomes among WCBA.

## 1 Introduction

Cancer has become a leading cause of morbidity and mortality among women worldwide, with profound implications for health systems and social development (1, 2). While much attention has been devoted to cancer in older women, the burden among women of childbearing age (WCBA, 15–49 years) remains under-recognized despite its substantial public health and socioeconomic consequences (3). Cancers occurring in this life stage not only threaten survival but also disrupt fertility, family stability, and workforce participation (4, 5). The United Nations and World Health Organization have emphasized addressing women’s health in this age group as integral to achieving Sustainable Development Goals, particularly those related to maternal health, gender equity, and non-communicable disease control (6, 7).

Northeast Asia (NEA)—comprising China (CHN), Japan (JPN), the Republic of Korea (KOR), Mongolia (MNG), the Democratic People’s Republic of Korea (PRK), and the Russian Federation (RUS)—represents a region of particular concern. Together, these countries account for a large and aging population with diverse sociopolitical contexts, rapid epidemiological transitions, and distinct health system capacities (8, 9). NEA is home to some of the highest global burdens of cancer, with lung, breast, and colorectal cancers ranking among the leading causes of cancer deaths (2, 10). Moreover, strong regional disparities exist: while Japan and Korea have advanced screening programs and relatively favorable outcomes, Mongolia and Democratic People’s Republic of Korea face persistent limitations in early detection and treatment access (11, 12). By examining NEA as a whole, this study provides insights into both high- and middle-income contexts, as well as opportunities for regional collaboration in cancer control.

The focus on WCBA aged 15–49 years reflects both biological and societal considerations. Recent epidemiological analyses have highlighted distinct cancer patterns among adolescents and young adult women, emphasizing the importance of examining cancers occurring during reproductive years (13). Biologically, this age group experiences unique hormonal and reproductive exposures—including menarche, parity, contraceptive use, and breastfeeding—that substantially influence cancer risk (14–16). Societally, cancers arising in these years can disproportionately disrupt fertility, family planning, and economic productivity, imposing intergenerational consequences (3, 4). Additionally, evidence suggests an increasing incidence of early-onset cancers globally, raising concern that lifestyle changes, obesity, and environmental exposures may accelerate cancer occurrence in younger cohorts (5). Thus, WCBA represent a critical population for cancer prevention, surveillance, and intervention.

We selected six cancers for analysis: breast, cervical, uterine, ovarian, CRC (colon and rectum), and TBL (tracheal, bronchus, and lung) cancers. The first four are female-specific or gynecologic cancers and collectively account for the majority of cancer burden among WCBA worldwide (3, 8). Breast cancer is the most common cancer among women globally, with rising incidence in Asia (17, 18). Analyses based on the Global Burden of Disease (GBD) dataset have confirmed a sustained upward trajectory in global breast cancer incidence and projected further increases through 2040, particularly in low- and middle-SDI regions (19). Cervical cancer, though preventable, remains a leading cause of premature cancer mortality in low- and middle-income settings (6, 20). Ovarian and uterine cancers, though less common, are associated with high lethality or rapid increases in incidence, respectively (16, 21). We additionally included colorectal and lung cancers, which, although not sex-specific, rank among the top causes of cancer burden in women in NEA and are projected to rise in younger populations globally (22, 23). Together, these six cancers provide a comprehensive view of the leading threats to women’s health during reproductive years.

Several studies have used publicly available datasets such as the Global Burden of Disease (GBD) study and GLOBOCAN to characterize the global or regional burden of cancers among women. For example, Ginsburg et al. (1) and Sung et al. (2) provided comprehensive overviews of female cancer incidence and mortality worldwide using GLOBOCAN data, while recent GBD analyses have examined temporal patterns of site-specific cancers, including breast, cervical, ovarian, and colorectal cancers, across different sociodemographic contexts (3, 24). However, these studies have largely focused on all-age female populations or individual cancer sites, and few have specifically evaluated the burden among WCBA. Moreover, the patterns in Northeast Asia remain underexplored despite the region’s distinct demographic and epidemiological transitions.

Large-scale comparative studies have also revealed persistent disparities in cancer burden by region, income level, and sex, as well as projected widening inequalities toward 2050 (25). Building on recent global assessments of female cancers, our study provides the first systematic analysis of temporal trends, mortality-to-incidence ratios (MIRs), and Bayesian age–period–cohort (BAPC) projections for six cancers among WCBA in NEA. By combining long-term trends (1990–2021) with future projections to 2050, this work offers critical evidence to guide tailored prevention, early detection, and policy strategies aimed at reducing the cancer burden among younger women in this high-priority region.

## 2 Methods

### 2.1 Study population and data collection

In this study, we analyzed data on six cancers affecting women from the Global Burden of Diseases, Injuries, and Risk Factors Study (GBD) 2021. Although cancers in women encompass a wide range of sites, including breast, cervical, ovarian, uterine, vulvar, vaginal, colorectal, and lung cancers, the present analysis focused on six cancers with the greatest burden among women of childbearing age (WCBA): breast cancer, cervical cancer, uterine cancer, ovarian cancer, colorectal cancer, and tracheal/bronchus/lung cancer. International Classification of Diseases (ICD) codes for these cancers were derived from the GBD 2021 cause mapping database (https://ghdx.healthdata.org/record/ihme-data/gbd-2021-cause-icd-code-mappings). Notably, according to the GBD definition, fallopian tube cancer was not included under ovarian cancer.

Following the World Health Organization (WHO) definition, WCBA was defined as women aged 15–49 years. We restricted our analysis to six countries in Northeast Asia (NEA)—China, Japan, the Republic of Korea, Mongolia, the Democratic People’s Republic of Korea, and the Russian Federation—given their large populations, epidemiological transitions, and unique heterogeneity in cancer control policies.

The GBD 2021, conducted by the Institute for Health Metrics and Evaluation (IHME) with contributions from over 11,500 collaborators across 164 countries, systematically assesses global health status and disease burden through extensive data collection, statistical modeling, and expert validation (26). Primary data sources include vital registration systems, cancer registries, household surveys, and hospital records, the details of which can be accessed through the GBD 2021 Data Input Sources Tool (https://ghdx.healthdata.org/gbd-2021/sources).

An overview of GBD data collection, modeling strategies, and dissemination processes has been described elsewhere and in the GBD methods appendices (https://www.healthdata.org/gbd/methods-appendices-2021/cancers). In brief, GBD applies standardized modeling frameworks to produce internally consistent estimates of incidence, mortality, years lived with disability (YLDs), years of life lost (YLLs), and disability-adjusted life years (DALYs) for 371 diseases and injuries across 204 countries and territories. For this study, we extracted cancer-specific numbers and age-standardized rates (ASRs) of incidence, mortality, and DALYs for the six cancers of interest in women aged 15–49 years from 1990 to 2021 using the GBD Results Tool (https://vizhub.healthdata.org/gbd-results/).

### 2.2 Statistical analysis

To assess how the relative burden among WCBA varied across cancer types and countries, we calculated the proportion of WCBA among all female cases by dividing the number of cases aged 15–49 years by the total number of female cases and multiplying by 100. The uncertainty intervals for the proportions were estimated using the logit-transformation method, as described by previous study (27). This approach stabilizes the variance of proportion data and provides approximate normality for the computation of standard errors. To evaluate temporal trends, we fitted a logistic regression model with calendar year as the explanatory variable. Details of the formula were in **Supplementary Text**.

The mortality-to-incidence ratio (MIR) is a practical indicator for evaluating cancer outcomes, often used to approximate case fatality and to assess the effectiveness of cancer surveillance and control programs, particularly cancer screening (28). MIR is conventionally calculated by dividing crude mortality by crude incidence rates or numbers of deaths by numbers of new cases (29). In this study, we calculated MIR for each cancer type and country as: $MIR(i,j)=\frac{Number\;of\;incidentcases(i,j)}{Number\;of\;deaths(i,j)}\times 100\%$ A higher MIR indicates poorer survival after diagnosis, while lower MIR suggests effective early detection and treatment. Following (25), we applied 46.6% as a reference threshold, which represents the global MIR for all cancers in 2022. A simple linear regression was used to explore the trend.

To enable comparisons across countries, cancers, and time, We calculated age-standardized rates (ASRs, per 100,000 population) for incidence, deaths, and DALYs using the direct standardization method, with the GBD standard population (30) as reference. The corresponding 95% confidence intervals (CIs) were estimated using exact methods implemented through the ageadjust.direct function in the epitools package in R (31). To evaluate temporal trends in ASRs, we calculated the estimated annual percentage change (EAPC). Details of the formula were in **Supplementary Text**.

To project future cancer burden among WCBA, we applied a Bayesian age–period–cohort (BAPC) model with well-calibrated probabilistic predictions. In this framework, all unknown parameters are treated as random variables with appropriate prior distributions. Smoothing priors and second-order random walks are commonly used to capture temporal patterns across age, period, and cohort dimensions (32). The BAPC model employs integrated nested Laplace approximation (INLA) to estimate posterior marginal distributions directly, thereby avoiding the computational challenges typically associated with Markov chain Monte Carlo (MCMC) sampling, such as mixing and convergence issues. This approach provides efficient, robust, and reliable inference for long-term projections (33). Projections of age-standardized incidence and mortality rates for six selected cancers were generated for each NEA country up to the year 2050 using this model.

*P* < 0.05 was considered statistically significant. All statistical analyses and graphical visualizations were performed using R software (version 4.4.2; R Foundation for Statistical Computing, Vienna, Austria).

## 3 Results

### 3.1 Trends in the proportion of WCBA among selected cancer incidence, deaths, and DALYs

**Figure 1:**
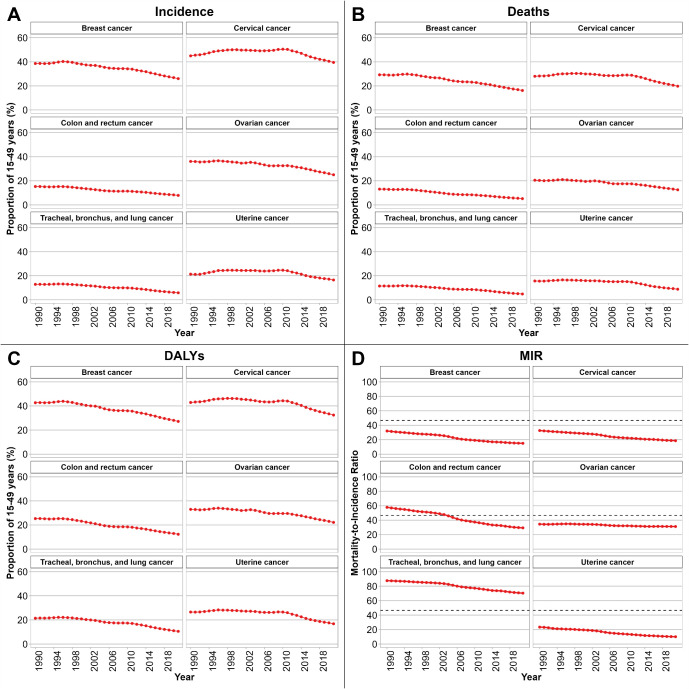
Temporal trends in the proportion of WCBA among selected cancer incidence **(A)**, deaths **(B)**, and DALYs **(C)**, 1990–2021. Patterns of MIR among WCBA by cancer type are shown in **(D)**. NEA, Northeast Asia. WCBA, women of childbearing age (15–49 years). DALYs, disability-adjusted life years. MIR, mortality-to-incidence ratio. uncertainty intervals for proportions were too narrow to be visible.

**Figures 1A–1C** depict temporal trends in the proportion of cancer incident cases, deaths, and DALYs among WCBA relative to total female cases in NEA from 1990 to 2021, stratified by cancer type. In **Figure 1A**, the proportion of incident cases in WCBA declined across all selected cancers, with the steepest decrease observed for TBL cancer ( $OR_{trend}$ = 0. 972, 95% CI: 0. 969 to 0. 976, **Supplementary Table S1**). In **Figure 1B**, the proportion of deaths in WCBA declined across all selected cancers, with the steepest decrease observed for CRC cancer ( $OR_{trend}$ = 0. 967, 95% CI: 0. 965 to 0. 969, **Supplementary Table S1**). In **Figure 1C**, the proportion of DALYs in WCBA declined across all selected cancers, with the steepest decrease observed for CRC cancer ( $OR_{trend}$ = 0. 971, 95% CI: 0. 969 to 0. 973, **Supplementary Table S1**). We also present in **Supplementary Figure S1** the temporal trends in the proportion of cancer incidence, deaths, and DALYs among WCBA relative to total female cases, stratified by country and cancer type.

### 3.2 Patterns of MIR among WCBA by cancer type and country

Specifically, we calculated MIR for six cancers among WCBA across six countries from 1990 to 2021, as shown in **Figure 1D**. An MIR threshold of 46.6% was applied as a reference point. CRC cancer exhibited the most rapid declining trend ( β = -0.010, 95% CI: -0.011 to -0.010, **Supplementary Table S2**). For TBL cancer, the MIR values were consistently above this threshold, although a declining trend was observed ( β = -0.006, 95% CI: -0.007 to -0.006, **Supplementary Table S2**). Additionally, we also calculated MIR for six cancers stratified by country as shown in **Supplementary Figure S1D**. MIR values were consistently below the 46.6% threshold for breast and cervical cancers in most countries, with notable downward trends observed in China, Japan, and the Republic of Korea. In contrast, CRC cancer and TBL cancer frequently showed MIR exceeding 46.6% in the early 1990s, although gradual declines were evident over time, particularly in China and the Republic of Korea. Ovarian cancer and uterine cancer exhibited MIR fluctuating around the reference threshold, with relatively stable patterns in Mongolia, the Democratic People’s Republic of Korea, and the Russian Federation.

### 3.3 Trends in incidence, deaths, and DALYs for selected cancers

**Table 1** summarizes the numbers, age-standardized rates (ASRs, per 100,000), and EAPCs six cancers among WCBA. For incidence, the ASRs of breast, cervical, and uterine cancers demonstrated increasing trends, with the most rapid rise observed in breast cancer (EAPC = 1.58%, 95% CI: 1.49 to 1.66). In contrast, the ASRs of ovarian cancer and TBL cancer declined over time, with the steepest decrease occurring in ovarian cancer (EAPC = -0.44%, 95% CI: -0.53 to -0.36). CRC cancer exhibited an upward trend in ASRs, although this was not statistically significant. For mortality, ASRs decreased across all selected cancers, with the sharpest decline observed in uterine cancer (EAPC = -2.38%, 95% CI: -2.68% to -2.09%). Similarly, for DALYs, ASRs declined for all selected cancers, with the steepest reduction observed in CRC cancer (EAPC = -2.30%, 95% CI: -2.49% to -2.12%). EAPCs by country and cancer type are provided in **Supplementary Table S3**.

**Table 1:** Incidence, deaths, and DALYs of selected cancers among WCBA in 1990 and 2021, and their estimated annual percentage changes from 1990 to 2021.

Cancer	Number of cases, 1990	Age-standardized rate per 100,000 population, 1990	Number of cases, 2021	Age-standardized rate per 100,000 population, 2021	EAPC, 1990-2021
Incidence					
Breast cancer	60133 (50893, 70596)	16.9 (16.8, 17.1)	145754 (113251, 182503)	28.6 (28.5, 28.8)	1.58 (1.49, 1.66)
Cervical cancer	38151 (31346, 46743)	10.4 (10.3, 10.5)	67777 (50952, 86318)	13.9 (13.8, 14.0)	1.40 (1.23, 1.56)
Colon and rectum cancer	20094 (16521, 24366)	5.6 (5.5, 5.7)	29409 (22247, 38322)	5.9 (5.8, 6.0)	0.02 (-0.19, 0.22)
Ovarian cancer	14125 (10110, 17632)	3.8 (3.7, 3.9)	16668 (12869, 21322)	3.5 (3.5, 3.6)	-0.44 (-0.53, -0.36)
Tracheal, bronchus, and lung cancer	14604 (11685, 18127)	4.1 (4.1, 4.2)	21839 (16621, 28052)	4.2 (4.2, 4.3)	-0.20 (-0.29, -0.10)
Uterine cancer	12390 (8690, 15271)	3.5 (3.4, 3.6)	22027 (16395, 29320)	4.3 (4.2, 4.4)	0.55 (0.31, 0.79)
Deaths					
Breast cancer	19275 (16158, 22872)	5.4 (5.4, 5.5)	21838 (16998, 27515)	4.3 (4.2, 4.3)	-1.18 (-1.32, -1.03)
Cervical cancer	12456 (10112, 15318)	3.5 (3.4, 3.5)	12606 (9536, 16048)	2.5 (2.4, 2.5)	-0.83 (-0.94, -0.71)
Colon and rectum cancer	11605 (9405, 14271)	3.2 (3.2, 3.3)	8635 (6533, 11248)	1.7 (1.7, 1.8)	-2.36 (-2.54, -2.18)
Ovarian cancer	4869 (3590, 6035)	1.4 (1.3, 1.4)	5198 (4012, 6620)	1.0 (1.0, 1.1)	-1.24 (-1.36, -1.12)
Tracheal, bronchus, and lung cancer	12797 (10218, 15868)	3.6 (3.6, 3.7)	15375 (11671, 19734)	3.0 (2.9, 3.0)	-0.97 (-1.10, -0.84)
Uterine cancer	2901 (1810, 3718)	0.8 (0.8, 0.9)	2222 (1641, 2928)	0.4 (0.4, 0.5)	-2.38 (-2.68, -2.09)
DALYs					
Breast cancer	990584 (828581, 1177986)	275.6 (275.0, 276.1)	1141640 (890991, 1437567)	226.0 (225.6, 226.4)	-1.01 (-1.14, -0.88)
Cervical cancer	642298 (521376, 790811)	176.2 (175.7, 176.6)	641899 (485659, 822318)	129.3 (128.9, 129.6)	-0.76 (-0.87, -0.64)
Colon and rectum cancer	600510 (484773, 739325)	163.3 (162.9, 163.7)	435918 (328350, 569619)	89.3 (89.1, 89.6)	-2.30 (-2.49, -2.12)
Ovarian cancer	252508 (183282, 313903)	69.5 (69.2, 69.8)	258696 (198853, 329446)	52.6 (52.4, 52.8)	-1.20 (-1.31, -1.09)
Tracheal, bronchus, and lung cancer	635619 (507576, 788304)	177.3 (176.9, 177.7)	732294 (556607, 941500)	144.7 (144.4, 145.1)	-1.00 (-1.13, -0.87)
Uterine cancer	149316 (92132, 191895)	41.6 (41.4, 41.8)	117101 (85989, 156342)	23.2 (23.1, 23.3)	-2.19 (-2.47, -1.91)

WCBA, women of childbearing age (15–49 years). DALYs, disability-adjusted life years.

### 3.4 Age distribution of WCBA burden across cancer types

**Figure 2:**
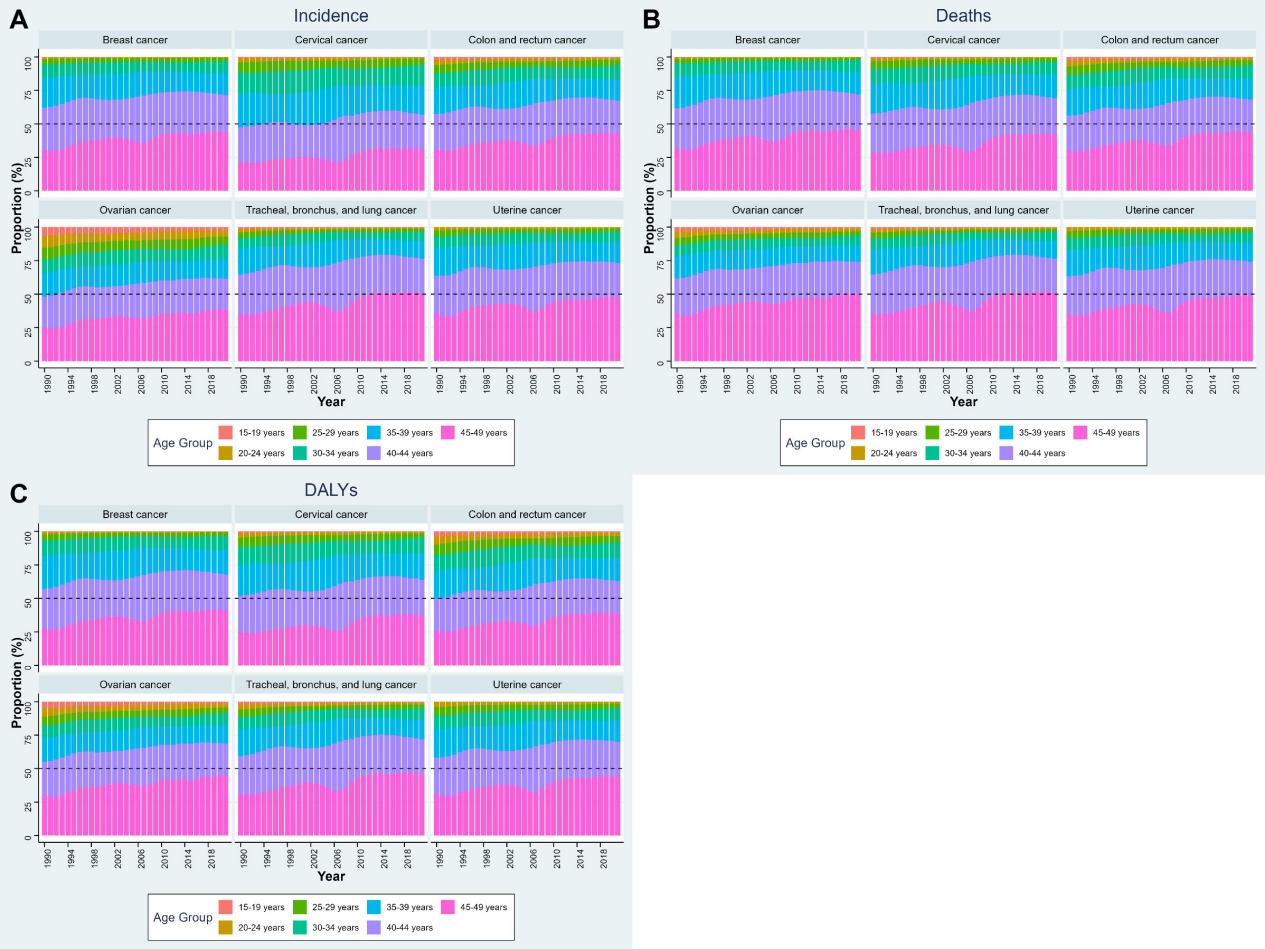
Age-specific composition of cancer burden among WCBA in NEA countries, 1990–2021. **(A)** Proportion of incidence, **(B)** proportion of deaths, and **(C)** proportion of DALYs, by 5-year age groups. Stacked bars indicate the relative contribution of each age subgroup (15–19, 20–24, 25–29, 30–34, 35–39, 40–44, and 45–49 years) to the total burden of each cancer type. NEA, Northeast Asia. WCBA, women of childbearing age (15–49 years). DALYs, disability-adjusted life years.

**Figure 2** illustrates the age-specific composition of cancer incidence, deaths, and DALYs among WCBA for six cancer sites across six NEA countries. Several consistent patterns were observed across outcomes. For incidence, the majority of cases were concentrated in women aged 40–44 years and 45–49 years, with these two groups together accounting for more than half of incident cases across all cancer types. This pattern was especially pronounced for breast, cervical, and colorectal cancers. For deaths, a similar age gradient was evident: the burden was disproportionately concentrated among women aged 45–49 years, followed by the 40–44 years group. The share of deaths attributable to women below 30 years remained minimal throughout the study period. For DALYs, the distribution mirrored incidence but emphasized the impact in younger reproductive ages. Although absolute contributions from the 15–29 years groups were small, they accounted for a larger share of DALYs than of deaths, reflecting the loss of life-years when cancers occur at younger ages. Nonetheless, the 40–44 and 45–49 years groups still contributed the majority of DALYs across all six cancers. The age-specific composition of cancer incidence, deaths, and DALYs by cancer type in each country is shown in **Supplementary Figure S2**.

### 3.5 Projections of incidence and mortality rates using BAPC

**Figure 3:**
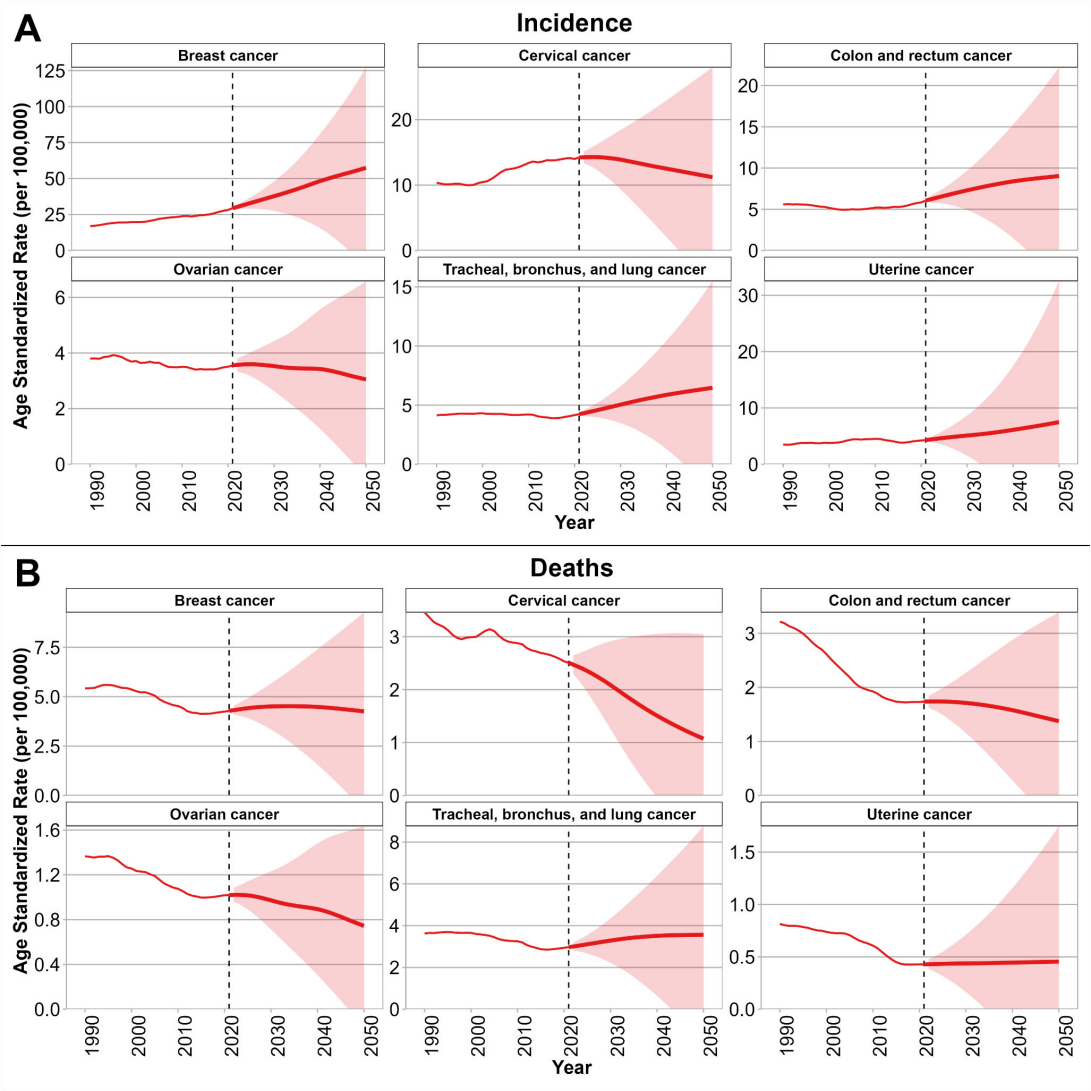
Projections of incidence **(A)** and mortality **(B)** rates for six selected cancers among WCBA in NEA through 2050 using the BAPC model. Solid red lines indicate observed and projected age-standardized rates (per 100,000), and shaded areas represent 95% uncertainty intervals. Vertical dashed lines mark the transition between observed data (1990–2021) and projections (2022–2050). NEA, Northeast Asia. WCBA, women of childbearing age (15–49 years). BAPC, Bayesian age–period–cohort. Note: Thin lines represent the observed period (1990–2021), and thick lines represent the projection period (2022–2050).

**Figures 3A, B** show the BAPC model projections of age-standardized incidence and death rates for six selected cancers among WCBA up to the year 2050. As shown in **Figure 3A**, incidence rates are projected to continue rising for breast, cervical, uterine, CRC, TBL cancers, with the steepest increases expected for breast and uterine cancers. In contrast, ovarian cancer incidence is projected to decline gradually. In **Figure 3B**, age-standardized mortality rates are projected to decrease for cervical, CRC, ovarian cancers, with the most pronounced reductions in cervical cancers. However, age-standardized mortality from breast, uterine, and TBL cancers is projected to remain relatively stable rather than decline substantially. Projections of age-standardized incidence and mortality by country are provided in **Supplementary Figure S3**. Briefly, age-standardized incidence of breast cancer is projected to keep rising trends in China, Mongolia, and the Democratic People’s Republic of Korea, while relatively stable patterns are expected in Japan, the Republic of Korea, and the Russian Federation. Age-standardized incidence of cervical cancer incidence is forecasted to continue declining in China, Japan, and the Republic of Korea, but will remain at comparatively high levels in Mongolia and the Democratic People’s Republic of Korea. Age-standardized mortality of breast cancer is projected to stabilize or decline in Japan, the Republic of Korea, and China, whereas increases are anticipated in Mongolia and the Democratic People’s Republic of Korea. Age-standardized mortality of cervical cancer deaths is expected to decline substantially in China, Japan, and the Republic of Korea, but remain high in Mongolia and the Democratic People’s Republic of Korea.

## 4 Discussion

In this study, we comprehensively examined temporal trends, mortality-to-incidence ratios), age-standardized rates, and conducted BAPC projections for six selected cancers among WCBA across six NEA countries. Previous studies using GBD and GLOBOCAN data have described global and regional cancer patterns among women, documenting rising burdens of breast and colorectal cancers and persistent inequities in cervical cancer outcomes (2, 3, 9, 24, 34). However, these assessments have largely focused on all-age female populations or single cancer sites. Our study extends this evidence base by specifically quantifying temporal trends, mortality-to-incidence ratios, and future projections among WCBA in NEA, a region characterized by rapid demographic transition and diverse health-system capacities. First, we observed that the proportion of cancer incidence, deaths, and DALYs occurring in WCBA relative to all female cases declined consistently across cancer types between 1990 and 2021. Second, MIR exhibited overall downward trends, with breast and cervical cancers remaining below the reference threshold, while CRC and TBL cancer cancers showed initially high but gradually declining MIRs, alongside marked cross-country heterogeneity. Third, ASR and EAPC analyses revealed distinct cancer-specific patterns: incidence increased most rapidly for breast, cervical, and uterine cancers, decreased for ovarian and TBL cancers, and remained relatively stable for CRC cancer, whereas mortality and DALYs declined for most cancers, with the steepest reductions in uterine, cervical, and CRC cancers. Finally, BAPC projections suggest a continued rise in breast and uterine cancer incidence through 2050, persistent declines in ovarian and cervical cancer in some countries, and divergent mortality trajectories, highlighting sustained disparities across NEA countries.

### 4.1 Declining relative burden of cancer among WCBA

Our first major finding was that the proportion of incident cases, deaths, and DALYs attributable to WCBA declined across all cancer types during 1990–2021. This relative decline should be interpreted in the context of demographic and epidemiological transitions in NEA. Population aging has accelerated markedly in countries such as Japan, the Republic of Korea, and China, leading to a growing share of cancers among postmenopausal women (24). The shifting age structure effectively reduces the relative proportion of WCBA, even if their absolute case counts remain substantial. Another explanation is that many cancers show steeper increases in incidence among older age groups compared to WCBA. For example, CRC and TBL cancers are more strongly associated with cumulative exposure to risk factors such as smoking, diet, and environmental pollution, which intensify with age (2, 22). Consequently, the proportion of cases among younger women declines relative to older cohorts. Improvements in prevention and early detection targeted at WCBA may also contribute to their declining share of cancer deaths and DALYs. For cervical cancer, widespread introduction of Pap smears, HPV DNA testing, and in some countries HPV vaccination has disproportionately benefited younger women (34, 35). Likewise, breast cancer awareness campaigns, earlier diagnostic practices, and advances in systemic therapies have reduced mortality rates among younger women in high-resource NEA settings (18, 36). However, this relative decline should not obscure the continued substantial impact of cancer among WCBA. These cancers strike women during economically and socially productive years, with downstream effects on fertility, family stability, and economic participation (4). Thus, health policies should continue to prioritize cancer prevention and control in this age group, while also responding to the rising burden among older women.

### 4.2 Cross-country and cancer-specific differences in MIR

The second key finding was that MIR patterns varied widely by cancer type and country. Breast and cervical cancers consistently showed MIR values below the 46.6% reference threshold, reflecting relatively favorable survival outcomes. This aligns with prior research showing that MIR can serve as a proxy indicator of healthcare system effectiveness and access to early detection and treatment (37, 38). Countries with organized screening programs and broad access to oncology care tend to have lower MIRs, as observed for breast and cervical cancers in Japan and the Republic of Korea (12, 39). By contrast, TBL cancers consistently exhibited MIR values above 46.6%, despite gradual declines. This underscores the persistent lethality of lung cancer in WCBA, where late-stage diagnosis is common and therapeutic options are often less effective than for breast or cervical cancer (10, 40). Low-dose computed tomography (LDCT) screening has demonstrated mortality reductions in randomized trials (41), but its implementation remains limited in many NEA countries due to cost, infrastructure, and concerns about radiation exposure. Moreover, tobacco exposure remains a major driver of lung cancer in the region, with worrying increases in smoking prevalence among younger women in parts of China and the Russian Federation (23, 42). Indoor and outdoor air pollution, including fine particulate matter (PM2.5), further compound risks (9). CRC cancer presented a more intermediate pattern, with MIR values exceeding the threshold in the early 1990s but declining thereafter, particularly in China and the Republic of Korea. This likely reflects gradual improvements in screening uptake (via fecal occult blood testing and colonoscopy) and treatment advances. In contrast, ovarian and uterine cancers exhibited MIR values fluctuating around the threshold, reflecting persistent challenges in early detection, as no highly effective population-level screening tools are currently available (16, 21). The heterogeneity in MIR trends highlights the dual importance of biological factors (e.g., tumor aggressiveness, screening feasibility) and health system performance (e.g., access to diagnostics, timely treatment). Persistently high MIRs for lung cancer underscore the urgent need for intensified tobacco control, air pollution mitigation, and consideration of cost-effective screening strategies in NEA.

### 4.3 Heterogeneous trends in age-standardized incidence and mortality rates

Our third key finding concerned the heterogeneous trends in age-standardized incidence and mortality rates across cancer types. Breast, cervical, and uterine cancers all demonstrated increasing incidence among WCBA, with breast cancer showing the steepest growth. This is consistent with previous study (3). This finding also aligns with recent GBD analyses reporting a 118.7% global increase in breast cancer cases among women aged 15–49 years from 1990 to 2021 (43). Rising incidence is likely attributable to shifts in reproductive behaviors—including delayed childbearing, fewer pregnancies, and reduced breastfeeding—as well as increasing prevalence of obesity, physical inactivity, and alcohol consumption (14). Despite rising incidence, breast cancer mortality rates among WCBA declined or stabilized, producing relatively low MIR values. This reflects improvements in mammographic screening, early-stage detection, and the adoption of modern systemic therapies, including endocrine, targeted, and immunotherapies (44). Cervical cancer incidence also increased in WCBA, albeit with declining mortality and DALYs. This paradox reflects enhanced detection due to the gradual implementation of population-level screening programs (35). Japan provides an instructive example: the temporary suspension of proactive HPV vaccination recommendations in 2013 led to a marked drop in coverage, which may have long-term consequences for incidence trends (45). In contrast, ovarian and TBL cancers exhibited declining incidence in WCBA. Declines in ovarian cancer may be linked to protective reproductive patterns such as increased contraceptive use and fewer ovulatory cycles, as well as improvements in surgical and perioperative care (16, 46). The decline in female TBL cancer incidence may partly reflect reduced smoking rates in Japan and Korea, as well as modest reductions in household air pollution from solid fuels in China. Nevertheless, incidence remains high in Mongolia and Russia, where tobacco consumption and air pollution remain prevalent (9). CRC cancer presented a mixed picture: incidence in WCBA increased modestly but was not statistically significant. This echoes findings from Western countries where colorectal cancer is increasingly diagnosed among adults under 50, though trends vary across Asia (2, 10). Diets high in processed meat, physical inactivity, and obesity are implicated in younger-onset colorectal cancer, while uptake of colonoscopy remains low in younger women across NEA (22). Mortality and DALYs declined across nearly all cancers, with uterine, cervical, and CRC cancers showing the steepest reductions. These declines signal progress in treatment and earlier diagnosis, reflecting expanding access to gynecologic oncology, improved surgical outcomes, and the integration of adjuvant therapies (21). However, the persistence of high mortality from TBL and ovarian cancers, despite declines in incidence, underscores the limits of current therapeutic strategies. These contrasting trends are consistent with global transition patterns reported in recent epidemiological analyses (47).

## 4 Projected future burden and regional disparities

Our fourth key finding comes from BAPC projections, which indicate continued heterogeneity in cancer trends among WCBA across NEA. Breast and uterine cancer incidence are projected to rise steeply through 2050. Projected disparities across NEA countries warrant emphasis. China is likely to experience continued increases in breast cancer incidence, while Japan and Korea may achieve stabilization through robust screening and treatment access. Mongolia and DPRK are forecast to face persistently high cervical and breast cancer burdens due to limited screening and vaccination infrastructure. Russia presents a mixed picture, with high lung cancer incidence and mortality likely to persist unless tobacco control and air quality interventions are strengthened. Overall, BAPC projections emphasize both opportunities and threats. On the one hand, substantial reductions in cervical and colorectal cancer mortality highlight the potential of prevention and early detection strategies. On the other, rising incidence of breast and uterine cancers, coupled with stagnating lung cancer mortality, signal areas where intensified action is urgently required. These findings align with the Global Cancer Observatory and WHO projections, which anticipate shifting burdens toward non-communicable, lifestyle-related cancers in younger women worldwide.

## 5 Limitations

Our study has several limitations that should be acknowledged. First, the estimation of incidence, mortality, and DALYs among WCBA was derived from the GBD 2021 data. Although the GBD provides comprehensive and standardized estimates, the accuracy of results depends heavily on the availability and quality of cancer registry data within each country. For countries with limited cancer surveillance infrastructure, such as Mongolia and the DPRK, incomplete or low-quality input data may have introduced uncertainties into the modeled estimates. Second, our analysis was restricted to six selected cancers—breast, cervical, uterine, ovarian, colorectal, and tracheal/bronchus/lung cancers—based on their high burden among WCBA. While this focus enhances comparability across countries and facilitates interpretation, it excludes other cancers (e.g., liver, thyroid, stomach) that may also contribute substantially to cancer burden in this age group in Northeast Asia. Third, cross-country differences in diagnostic practices, screening uptake, and treatment protocols may have influenced comparability of trends. For example, variations in mammography and Pap smear coverage, the timing of HPV vaccination introduction, or differences in lung cancer screening with low-dose computed tomography may contribute to observed heterogeneity beyond underlying epidemiological patterns. Fourth, the interpretation of temporal trends based on ORs, EAPCs, and MIRs assumes a degree of linearity and stability in measurement. However, these indicators may not fully capture nonlinear dynamics, abrupt policy changes, or emerging risk factors that could alter trajectories in the short term. Finally, projections from the BAPC model inherently rely on the assumption that past trends will extend into the future. Unanticipated advances in prevention (e.g., widespread HPV vaccination, novel therapeutics) or changes in population behaviors (e.g., smoking, fertility patterns, obesity prevalence) may lead to deviations from the projected trajectories. Thus, while the projections provide valuable insights into potential future burdens, they should be interpreted cautiously.

## 6 Conclusions and policy implications

In conclusion, this study provides a comprehensive assessment of temporal trends, mortality-to-incidence ratios, age-standardized rates, and future projections of six selected cancers among WCBA across six NEA countries from 1990 to 2021. We found that the proportion of cancer burden in WCBA relative to all females has declined, yet substantial heterogeneity exists across cancer types and countries. Breast and uterine cancers continue to rise in incidence, while cervical and colorectal cancers show encouraging declines in mortality and DALYs, reflecting the impact of screening and treatment improvements. TBL cancer remains a critical concern, with persistently high MIRs underscoring gaps in prevention and early detection. Projections to 2050 highlight ongoing increases in breast and uterine cancer incidence and persistent disparities between high-resource and resource-limited settings. These findings emphasize the urgent need for targeted prevention, expanded screening and vaccination programs, improved treatment accessibility, and cross-country collaboration to reduce the cancer burden among WCBA in Northeast Asia.

## Data Availability

Publicly available datasets were analyzed in this study. This data can be found here: https://ghdx.healthdata.org/gbd-2021.
