# Supplementary material for: Temporal trends and future projections of six major cancers among women of childbearing age in northeast Asia: analysis of data from the global burden of disease study 2021
Source: Front Oncol. 2025 Nov 28;15:1716310. doi: 10.3389/fonc.2025.1716310 (PMC12698415; doi:10.3389/fonc.2025.1716310)
Supplement: Supplementary file 1 [file DataSheet1.docx]

Supplementary To

Temporal trends and future projections of six major cancers mmong women of childbearing age in northeast Asia: analysis of data from the global burden of disease study 2021

Liang Jingqian1, Huai Baogeng2, Yang Zeping3, Zhang Shan4, Cui Haoliang4, Zhang Jianyi4, Liu Deshan5*

1 Jinan Zhangqiu District Hospital of Traditional Chinese Medicine, Jinan 250200, China.

2 First Clinical Medical College, Shandong University of Traditional Chinese Medicine, Jinan, P. R. China.

3 Vanke School of Public Health, Tsinghua University, Beijing 100084, China.

4 School of Public Health, Peking University, Beijing 100191, China.

5 Department of Traditional Chinese Medicine, Qilu Hospital of Shandong University, Jinan, Shandong 250012, China.

* Corresponding author

Email: liudeshan_doc@163.com (D. Liu)

Table S1. Temporal trends in the proportion of WCBA among cancer incidence, mortality, and DALYs by cancer type

| Cancer | Incidence | |  | Deaths | |  | DALYs | |
| --- | --- | --- | --- | --- | --- | --- | --- | --- |
|  | OR (95% CI) | P |  | OR (95% CI) | P |  | OR (95% CI) | P |
| Breast cancer | 0.981 (0.979, 0.983) | <0.001 |  | 0.975 (0.973, 0.977) | <0.001 |  | 0.977 (0.975, 0.979) | <0.001 |
| Cervical cancer | 0.994 (0.989, 0.998) | 0.006 |  | 0.987 (0.983, 0.991) | <0.001 |  | 0.986 (0.982, 0.990) | <0.001 |
| Colon and rectum cancer | 0.976 (0.975, 0.978) | <0.001 |  | 0.967 (0.965, 0.969) | <0.001 |  | 0.971 (0.969, 0.973) | <0.001 |
| Ovarian cancer | 0.985 (0.982, 0.987) | <0.001 |  | 0.982 (0.980, 0.985) | <0.001 |  | 0.983 (0.981, 0.986) | <0.001 |
| Tracheal, bronchus, and lung cancer | 0.972 (0.969, 0.976) | <0.001 |  | 0.970 (0.967, 0.974) | <0.001 |  | 0.973 (0.970, 0.977) | <0.001 |
| Uterine cancer | 0.991 (0.986, 0.996) | 0.001 |  | 0.980 (0.976, 0.985) | <0.001 |  | 0.982 (0.978, 0.987) | <0.001 |

WCBA, women of childbearing age (15-49 years). DALYs, disability-adjusted life years.

Table S2. Temporal trend of MIR by cancer type

| Cancer | $\beta$ (95% CI) | P |
| --- | --- | --- |
| Breast cancer | -0.006 (-0.006, -0.006) | <0.001 |
| Cervical cancer | -0.005 (-0.005, -0.005) | <0.001 |
| Colon and rectum cancer | -0.010 (-0.011, -0.010) | <0.001 |
| Ovarian cancer | -0.001 (-0.002, -0.001) | <0.001 |
| Tracheal, bronchus, and lung cancer | -0.006 (-0.007, -0.006) | <0.001 |
| Uterine cancer | -0.005 (-0.005, -0.004) | <0.001 |

WCBA, women of childbearing age (15-49 years). MIR, mortality-to-incidence ratio.

Table S3. Number and age-standardized rate of incidence, deaths, and DALYs of selected cancers among WCBA in 1990 and 2021, and their estimated annual percentage changes from 1990 to 2021, stratified by NEA countries.

| Cancer | Country | Number of cases, 1990 | Age-standardized  rate per 100,000  population, 1990 | Number of cases, 2021 | Age-standardized  rate per 100,000  population, 2021 | EAPC, 1990-2021 |
| --- | --- | --- | --- | --- | --- | --- |
| Incidence | |  |  |  |  |  |
| Breast cancer | CHN | 36689 (29142, 45383) | 13.8 (13.7, 14.0) | 110371 (82543, 142345) | 27.3 (27.1, 27.4) | 2.16 (2.08, 2.24) |
| Breast cancer | JPN | 11234 (10286, 12123) | 29.2 (28.7, 29.8) | 14133 (12903, 15317) | 40.5 (39.9, 41.2) | 0.93 (0.57, 1.28) |
| Breast cancer | KOR | 1374 (1155, 1636) | 12.4 (11.8, 13.1) | 5620 (4508, 6854) | 36.4 (35.4, 37.4) | 3.85 (3.46, 4.25) |
| Breast cancer | MNG | 19 (15, 23) | 5.5 (3.3, 8.8) | 78 (60, 97) | 8.4 (6.7, 10.6) | 1.39 (1.24, 1.55) |
| Breast cancer | PRK | 706 (451, 1061) | 13.5 (12.5, 14.5) | 1629 (1059, 2382) | 22.0 (20.9, 23.1) | 1.80 (1.66, 1.94) |
| Breast cancer | RUS | 10111 (9844, 10370) | 27.4 (26.8, 27.9) | 13923 (12178, 15508) | 31.1 (30.6, 31.6) | 0.17 (-0.06, 0.39) |
| Cervical cancer | CHN | 28244 (22277, 35813) | 10.3 (10.1, 10.4) | 49796 (35518, 65634) | 12.7 (12.6, 12.9) | 1.24 (1.03, 1.44) |
| Cervical cancer | JPN | 3254 (3087, 3475) | 9.0 (8.7, 9.3) | 4452 (4074, 4863) | 14.4 (14.0, 14.8) | 1.83 (1.57, 2.10) |
| Cervical cancer | KOR | 1681 (1386, 2003) | 14.8 (14.1, 15.5) | 1429 (1125, 1855) | 10.2 (9.7, 10.8) | -1.09 (-1.34, -0.84) |
| Cervical cancer | MNG | 66 (47, 90) | 18.1 (13.9, 23.2) | 154 (113, 199) | 16.6 (14.1, 19.5) | -0.40 (-0.64, -0.17) |
| Cervical cancer | PRK | 711 (479, 1037) | 13.6 (12.6, 14.6) | 1280 (820, 1873) | 17.6 (16.6, 18.6) | 0.99 (0.88, 1.10) |
| Cervical cancer | RUS | 4195 (4071, 4324) | 10.8 (10.4, 11.1) | 10666 (9301, 11894) | 24.9 (24.4, 25.4) | 2.85 (2.63, 3.07) |
| CRC caner | CHN | 14230 (11034, 18060) | 5.2 (5.1, 5.3) | 22767 (16569, 30498) | 5.7 (5.7, 5.8) | 0.11 (-0.14, 0.36) |
| CRC caner | JPN | 3083 (2947, 3229) | 7.9 (7.6, 8.2) | 2723 (2468, 3015) | 7.8 (7.5, 8.1) | 0.01 (-0.10, 0.12) |
| CRC caner | KOR | 521 (415, 634) | 4.7 (4.3, 5.1) | 995 (753, 1322) | 6.5 (6.1, 6.9) | 1.24 (1.01, 1.48) |
| CRC caner | MNG | 10 (7, 12) | 2.6 (1.2, 5.1) | 23 (17, 30) | 2.5 (1.6, 3.9) | -0.35 (-0.59, -0.11) |
| CRC caner | PRK | 206 (133, 330) | 3.9 (3.3, 4.4) | 314 (176, 564) | 4.2 (3.8, 4.7) | 0.36 (0.25, 0.48) |
| CRC caner | RUS | 2045 (1985, 2101) | 5.6 (5.3, 5.8) | 2587 (2263, 2892) | 5.9 (5.6, 6.1) | 0.19 (-0.04, 0.42) |
| Ovarian cancer | CHN | 8588 (5020, 11725) | 3.0 (2.9, 3.1) | 11339 (8279, 15200) | 3.0 (3.0, 3.1) | -0.16 (-0.26, -0.06) |
| Ovarian cancer | JPN | 1892 (1804, 1983) | 5.2 (4.9, 5.4) | 1551 (1465, 1658) | 4.9 (4.7, 5.2) | -0.15 (-0.39, 0.10) |
| Ovarian cancer | KOR | 367 (235, 427) | 3.1 (2.8, 3.5) | 574 (416, 703) | 4.0 (3.7, 4.4) | 0.80 (0.68, 0.93) |
| Ovarian cancer | MNG | 15 (10, 23) | 3.9 (2.1, 6.7) | 41 (28, 56) | 4.6 (3.3, 6.3) | 0.41 (0.24, 0.57) |
| Ovarian cancer | PRK | 235 (134, 352) | 4.3 (3.8, 4.9) | 452 (281, 672) | 6.3 (5.8, 7.0) | 1.29 (1.23, 1.34) |
| Ovarian cancer | RUS | 3027 (2907, 3122) | 8.3 (8.0, 8.6) | 2711 (2400, 3034) | 6.4 (6.2, 6.7) | -0.93 (-1.09, -0.77) |
| TBL cancer | CHN | 12346 (9626, 15660) | 4.7 (4.7, 4.8) | 19707 (14839, 25509) | 4.8 (4.7, 4.9) | -0.28 (-0.39, -0.16) |
| TBL cancer | JPN | 988 (943, 1033) | 2.5 (2.4, 2.7) | 648 (595, 695) | 1.8 (1.7, 2.0) | -1.25 (-1.61, -0.88) |
| TBL cancer | KOR | 323 (267, 373) | 3.0 (2.6, 3.3) | 395 (317, 496) | 2.5 (2.3, 2.8) | -0.35 (-0.52, -0.18) |
| TBL cancer | MNG | 11 (8, 14) | 3.1 (1.5, 5.7) | 18 (13, 23) | 2.0 (1.2, 3.2) | -2.14 (-2.51, -1.77) |
| TBL cancer | PRK | 199 (131, 287) | 3.7 (3.2, 4.3) | 278 (161, 444) | 3.7 (3.2, 4.1) | -0.01 (-0.10, 0.08) |
| TBL cancer | RUS | 737 (710, 761) | 2.0 (1.9, 2.2) | 793 (696, 885) | 1.8 (1.7, 1.9) | -0.59 (-0.73, -0.45) |
| Uterine cancer | CHN | 7609 (4291, 10082) | 2.9 (2.8, 3.0) | 14312 (9842, 20370) | 3.5 (3.5, 3.6) | 0.62 (0.21, 1.03) |
| Uterine cancer | JPN | 772 (715, 832) | 2.0 (1.9, 2.2) | 1967 (1794, 2155) | 5.5 (5.3, 5.8) | 3.79 (3.51, 4.07) |
| Uterine cancer | KOR | 228 (101, 323) | 2.1 (1.8, 2.4) | 380 (226, 562) | 2.5 (2.2, 2.7) | 1.21 (0.74, 1.69) |
| Uterine cancer | MNG | 7 (4, 11) | 2.0 (0.7, 4.2) | 24 (15, 36) | 2.6 (1.7, 3.9) | 0.92 (0.72, 1.12) |
| Uterine cancer | PRK | 176 (102, 269) | 3.3 (2.9, 3.9) | 360 (194, 574) | 4.8 (4.3, 5.3) | 1.33 (1.19, 1.46) |
| Uterine cancer | RUS | 3598 (3477, 3752) | 9.9 (9.6, 10.2) | 4985 (4324, 5622) | 11.1 (10.8, 11.4) | 0.19 (-0.21, 0.58) |
| Deaths |  |  |  |  |  |  |
| Breast cancer | CHN | 13567 (10747, 16803) | 5.1 (5.0, 5.2) | 16439 (12282, 21420) | 4.0 (4.0, 4.1) | -1.11 (-1.24, -0.98) |
| Breast cancer | JPN | 1792 (1749, 1836) | 4.6 (4.4, 4.8) | 1459 (1417, 1494) | 4.1 (3.9, 4.3) | -0.65 (-0.89, -0.41) |
| Breast cancer | KOR | 424 (363, 503) | 3.9 (3.5, 4.3) | 616 (506, 734) | 3.9 (3.6, 4.3) | 0.10 (0.01, 0.18) |
| Breast cancer | MNG | 10 (8, 12) | 2.9 (1.4, 5.5) | 27 (21, 33) | 2.9 (1.9, 4.3) | -0.24 (-0.44, -0.04) |
| Breast cancer | PRK | 305 (194, 461) | 5.8 (5.2, 6.5) | 501 (326, 720) | 6.7 (6.1, 7.3) | 0.55 (0.48, 0.61) |
| Breast cancer | RUS | 3177 (3096, 3258) | 8.6 (8.3, 8.9) | 2795 (2446, 3113) | 6.2 (6.0, 6.5) | -1.52 (-1.72, -1.31) |
| Cervical cancer | CHN | 10020 (7890, 12628) | 3.8 (3.7, 3.8) | 9212 (6630, 12142) | 2.3 (2.2, 2.3) | -1.30 (-1.45, -1.15) |
| Cervical cancer | JPN | 549 (534, 565) | 1.4 (1.3, 1.6) | 594 (574, 613) | 1.8 (1.6, 1.9) | 0.82 (0.58, 1.06) |
| Cervical cancer | KOR | 435 (359, 513) | 4.0 (3.6, 4.4) | 187 (149, 243) | 1.2 (1.1, 1.4) | -3.83 (-4.18, -3.48) |
| Cervical cancer | MNG | 27 (19, 37) | 7.6 (5.0, 11.3) | 44 (33, 58) | 4.8 (3.5, 6.5) | -1.88 (-2.17, -1.60) |
| Cervical cancer | PRK | 253 (170, 366) | 4.8 (4.2, 5.4) | 368 (230, 535) | 4.9 (4.5, 5.5) | 0.19 (0.12, 0.27) |
| Cervical cancer | RUS | 1172 (1139, 1210) | 3.1 (2.9, 3.3) | 2201 (1919, 2458) | 5.0 (4.8, 5.2) | 1.49 (1.21, 1.77) |
| CRC caner | CHN | 9022 (6988, 11475) | 3.3 (3.3, 3.4) | 6667 (4832, 8938) | 1.7 (1.6, 1.7) | -2.59 (-2.82, -2.36) |
| CRC caner | JPN | 1043 (1021, 1068) | 2.7 (2.5, 2.9) | 609 (592, 627) | 1.7 (1.6, 1.9) | -1.46 (-1.59, -1.33) |
| CRC caner | KOR | 303 (240, 368) | 2.7 (2.4, 3.1) | 231 (176, 310) | 1.5 (1.3, 1.7) | -2.03 (-2.18, -1.87) |
| CRC caner | MNG | 7 (6, 10) | 2.0 (0.8, 4.3) | 14 (10, 18) | 1.5 (0.8, 2.7) | -1.22 (-1.46, -0.99) |
| CRC caner | PRK | 135 (87, 223) | 2.5 (2.1, 3.0) | 163 (91, 290) | 2.2 (1.9, 2.6) | -0.47 (-0.52, -0.41) |
| CRC caner | RUS | 1096 (1064, 1127) | 3.0 (2.8, 3.2) | 952 (832, 1064) | 2.2 (2.0, 2.3) | -1.23 (-1.41, -1.05) |
| Ovarian cancer | CHN | 2913 (1761, 3975) | 1.1 (1.0, 1.1) | 3532 (2553, 4735) | 0.9 (0.8, 0.9) | -1.00 (-1.12, -0.89) |
| Ovarian cancer | JPN | 720 (703, 737) | 1.9 (1.7, 2.0) | 468 (455, 482) | 1.3 (1.2, 1.5) | -1.04 (-1.16, -0.91) |
| Ovarian cancer | KOR | 131 (90, 150) | 1.2 (1.0, 1.4) | 171 (125, 209) | 1.1 (0.9, 1.3) | -0.24 (-0.41, -0.06) |
| Ovarian cancer | MNG | 6 (4, 9) | 1.7 (0.6, 3.8) | 16 (11, 21) | 1.7 (1.0, 2.9) | -0.03 (-0.22, 0.15) |
| Ovarian cancer | PRK | 55 (32, 85) | 1.0 (0.8, 1.3) | 94 (58, 142) | 1.3 (1.0, 1.5) | 0.69 (0.62, 0.76) |
| Ovarian cancer | RUS | 1045 (1001, 1079) | 2.9 (2.7, 3.1) | 919 (810, 1032) | 2.1 (1.9, 2.2) | -1.22 (-1.39, -1.05) |
| TBL cancer | CHN | 11043 (8622, 13944) | 4.2 (4.2, 4.3) | 13999 (10527, 18083) | 3.4 (3.3, 3.5) | -1.09 (-1.23, -0.94) |
| TBL cancer | JPN | 667 (648, 686) | 1.7 (1.6, 1.8) | 327 (317, 338) | 0.9 (0.8, 1.0) | -2.41 (-2.62, -2.20) |
| TBL cancer | KOR | 275 (227, 317) | 2.5 (2.2, 2.8) | 205 (167, 252) | 1.3 (1.1, 1.5) | -2.19 (-2.31, -2.07) |
| TBL cancer | MNG | 10 (8, 13) | 2.8 (1.3, 5.4) | 16 (12, 21) | 1.8 (1.0, 2.9) | -2.23 (-2.59, -1.86) |
| TBL cancer | PRK | 184 (118, 269) | 3.4 (3.0, 4.0) | 247 (143, 393) | 3.3 (2.9, 3.7) | -0.14 (-0.23, -0.06) |
| TBL cancer | RUS | 618 (596, 639) | 1.7 (1.6, 1.9) | 580 (505, 648) | 1.3 (1.2, 1.4) | -1.09 (-1.20, -0.98) |
| Uterine cancer | CHN | 2255 (1231, 3005) | 0.9 (0.8, 0.9) | 1597 (1110, 2201) | 0.4 (0.4, 0.4) | -2.81 (-3.18, -2.44) |
| Uterine cancer | JPN | 109 (107, 112) | 0.3 (0.2, 0.3) | 160 (155, 165) | 0.4 (0.4, 0.5) | 1.86 (1.69, 2.03) |
| Uterine cancer | KOR | 58 (26, 84) | 0.5 (0.4, 0.7) | 32 (19, 47) | 0.2 (0.1, 0.3) | -2.72 (-3.40, -2.03) |
| Uterine cancer | MNG | 2 (1, 4) | 0.7 (0.1, 2.4) | 5 (3, 7) | 0.5 (0.2, 1.3) | -1.21 (-1.43, -1.00) |
| Uterine cancer | PRK | 35 (20, 53) | 0.7 (0.5, 0.9) | 53 (29, 85) | 0.7 (0.5, 0.9) | 0.24 (0.18, 0.30) |
| Uterine cancer | RUS | 441 (427, 460) | 1.2 (1.1, 1.3) | 375 (326, 424) | 0.8 (0.8, 0.9) | -1.66 (-1.97, -1.34) |
| DALYs |  |  |  |  |  |  |
| Breast cancer | CHN | 699470 (554281, 866933) | 260.2 (259.6, 260.8) | 861867 (648610, 1119103) | 214.2 (213.8, 214.7) | -0.94 (-1.06, -0.81) |
| Breast cancer | JPN | 93340 (89927, 96903) | 244.1 (242.6, 245.7) | 78164 (74117, 82299) | 225.3 (223.7, 227.0) | -0.49 (-0.72, -0.26) |
| Breast cancer | KOR | 22102 (18988, 26132) | 198.9 (196.2, 201.5) | 33110 (27033, 39822) | 215.5 (213.1, 217.9) | 0.33 (0.23, 0.43) |
| Breast cancer | MNG | 505 (397, 625) | 145.4 (132.7, 159.0) | 1353 (1050, 1650) | 146.1 (138.4, 154.2) | -0.20 (-0.39, -0.01) |
| Breast cancer | PRK | 15124 (9625, 22659) | 289.2 (284.6, 293.9) | 24893 (16274, 35792) | 336.0 (331.8, 340.3) | 0.58 (0.51, 0.64) |
| Breast cancer | RUS | 160044 (155362, 164735) | 428.9 (426.8, 431.0) | 142255 (123905, 158901) | 318.7 (317.1, 320.4) | -1.40 (-1.59, -1.21) |
| Cervical cancer | CHN | 516820 (407042, 652148) | 190.2 (189.6, 190.7) | 467647 (335981, 621267) | 117.4 (117.1, 117.8) | -1.23 (-1.38, -1.09) |
| Cervical cancer | JPN | 28216 (27290, 29237) | 75.9 (75.0, 76.8) | 30551 (29361, 31896) | 93.4 (92.3, 94.5) | 0.87 (0.63, 1.12) |
| Cervical cancer | KOR | 22514 (18700, 26510) | 202.1 (199.4, 204.8) | 9798 (7926, 12709) | 66.9 (65.5, 68.3) | -3.62 (-3.97, -3.26) |
| Cervical cancer | MNG | 1362 (986, 1874) | 380.9 (360.4, 402.3) | 2224 (1650, 2881) | 241.4 (231.4, 251.7) | -1.85 (-2.12, -1.57) |
| Cervical cancer | PRK | 12745 (8607, 18319) | 241.8 (237.6, 246.1) | 18311 (11515, 26561) | 249.3 (245.7, 253.0) | 0.18 (0.11, 0.25) |
| Cervical cancer | RUS | 60640 (58750, 62722) | 158.2 (156.9, 159.5) | 113367 (99227, 127003) | 261.1 (259.6, 262.6) | 1.56 (1.26, 1.86) |
| CRC caner | CHN | 471929 (364596, 600389) | 169.5 (169.0, 170.0) | 338343 (244200, 455143) | 87.3 (87.0, 87.6) | -2.54 (-2.77, -2.30) |
| CRC caner | JPN | 51265 (50031, 52597) | 134.5 (133.3, 135.7) | 30273 (29262, 31326) | 89.5 (88.5, 90.6) | -1.36 (-1.49, -1.24) |
| CRC caner | KOR | 15571 (12532, 18577) | 138.1 (135.9, 140.3) | 11603 (8863, 15537) | 77.0 (75.6, 78.5) | -1.94 (-2.11, -1.77) |
| CRC caner | MNG | 379 (288, 495) | 101.7 (91.3, 112.9) | 707 (519, 910) | 77.6 (72.0, 83.6) | -1.19 (-1.42, -0.95) |
| CRC caner | PRK | 6816 (4391, 11146) | 127.3 (124.3, 130.4) | 8058 (4497, 14300) | 110.4 (108.0, 112.9) | -0.48 (-0.53, -0.42) |
| CRC caner | RUS | 54551 (52935, 56122) | 147.4 (146.2, 148.7) | 46934 (41010, 52403) | 107.7 (106.7, 108.7) | -1.17 (-1.35, -0.99) |
| Ovarian cancer | CHN | 153874 (91308, 209936) | 55.8 (55.5, 56.1) | 176403 (126934, 236355) | 45.1 (44.9, 45.4) | -0.98 (-1.10, -0.86) |
| Ovarian cancer | JPN | 35607 (34720, 36462) | 94.8 (93.8, 95.8) | 22990 (22319, 23656) | 68.9 (68.0, 69.9) | -1.01 (-1.14, -0.87) |
| Ovarian cancer | KOR | 6973 (4616, 8005) | 60.7 (59.3, 62.1) | 8476 (6233, 10253) | 56.6 (55.4, 57.9) | -0.30 (-0.48, -0.11) |
| Ovarian cancer | MNG | 320 (211, 469) | 84.7 (75.3, 95.0) | 795 (553, 1070) | 88.0 (82.0, 94.4) | -0.03 (-0.20, 0.15) |
| Ovarian cancer | PRK | 2863 (1625, 4379) | 53.0 (51.1, 55.0) | 4799 (2986, 7254) | 65.4 (63.6, 67.3) | 0.70 (0.64, 0.77) |
| Ovarian cancer | RUS | 52870 (50801, 54652) | 145.2 (144.0, 146.5) | 45233 (39828, 50858) | 104.3 (103.3, 105.3) | -1.23 (-1.40, -1.06) |
| TBL cancer | CHN | 550506 (429913, 694977) | 207.0 (206.4, 207.6) | 667020 (502305, 863200) | 165.2 (164.8, 165.6) | -1.11 (-1.25, -0.96) |
| TBL cancer | JPN | 31689 (30806, 32645) | 82.2 (81.3, 83.2) | 15409 (14906, 15900) | 44.4 (43.7, 45.2) | -2.37 (-2.57, -2.16) |
| TBL cancer | KOR | 13871 (11579, 16003) | 124.2 (122.1, 126.3) | 9768 (7931, 11980) | 63.5 (62.2, 64.8) | -2.24 (-2.37, -2.12) |
| TBL cancer | MNG | 523 (389, 677) | 140.2 (128.0, 153.3) | 796 (598, 1019) | 87.5 (81.5, 93.9) | -2.22 (-2.59, -1.85) |
| TBL cancer | PRK | 8837 (5795, 12791) | 166.0 (162.5, 169.5) | 11672 (6769, 18604) | 155.7 (152.9, 158.6) | -0.16 (-0.25, -0.08) |
| TBL cancer | RUS | 30193 (29095, 31210) | 82.6 (81.7, 83.5) | 27630 (24098, 30798) | 62.7 (62.0, 63.5) | -1.12 (-1.24, -1.01) |
| Uterine cancer | CHN | 115737 (62140, 154752) | 43.3 (43.0, 43.6) | 83645 (57606, 117196) | 20.9 (20.7, 21.0) | -2.61 (-2.98, -2.25) |
| Uterine cancer | JPN | 5537 (5348, 5747) | 14.4 (14.1, 14.8) | 8414 (8012, 8836) | 23.6 (23.1, 24.1) | 2.04 (1.86, 2.21) |
| Uterine cancer | KOR | 2936 (1275, 4201) | 27.1 (26.1, 28.1) | 1695 (1007, 2473) | 11.0 (10.5, 11.6) | -2.38 (-3.05, -1.70) |
| Uterine cancer | MNG | 118 (69, 191) | 33.3 (27.4, 40.1) | 238 (144, 358) | 25.9 (22.7, 29.5) | -1.14 (-1.34, -0.93) |
| Uterine cancer | PRK | 1768 (1012, 2653) | 33.3 (31.7, 34.9) | 2707 (1458, 4260) | 36.3 (34.9, 37.7) | 0.29 (0.23, 0.34) |
| Uterine cancer | RUS | 23219 (22289, 24351) | 63.1 (62.2, 63.9) | 20401 (17762, 23221) | 45.8 (45.2, 46.5) | -1.47 (-1.78, -1.16) |

NEA, Northeast Asia. WCBA, women of childbearing age (15-49 years). DALYs, disability-adjusted life years. MIR, mortality-to-incidence ratio. TBL cancer, tracheal, bronchus, and lung cancer. CRC caner, colon and rectum cancer. CHN, China. JPN, Japan. KOR, Republic of Korea. MNG, Mongolia. PRK, Democratic People's Republic of Korea. RUS, Russian Federation.


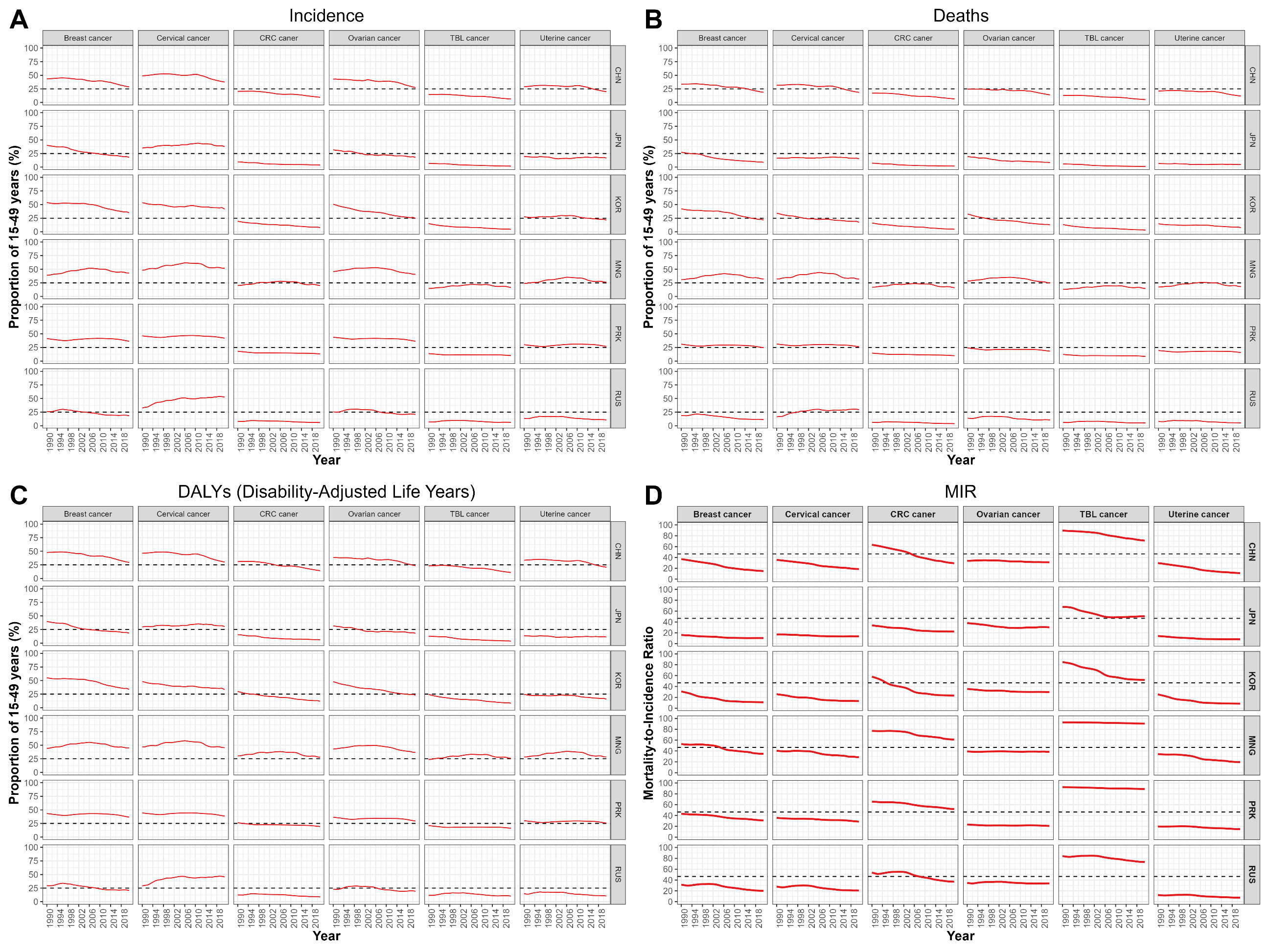


Figure S1. Temporal trends in the proportion of WCBA among selected cancer incidence (A), deaths (B), and DALYs (C) stratified by countries, 1990–2021. Patterns of MIR among WCBA by cancer type and country are shown in (D). NEA, Northeast Asia. WCBA, women of childbearing age (15-49 years). DALYs, disability-adjusted life years. MIR, mortality-to-incidence ratio. TBL cancer, tracheal, bronchus, and lung cancer. CRC caner, colon and rectum cancer. CHN, China. JPN, Japan. KOR, Republic of Korea. MNG, Mongolia. PRK, Democratic People's Republic of Korea. RUS, Russian Federation.


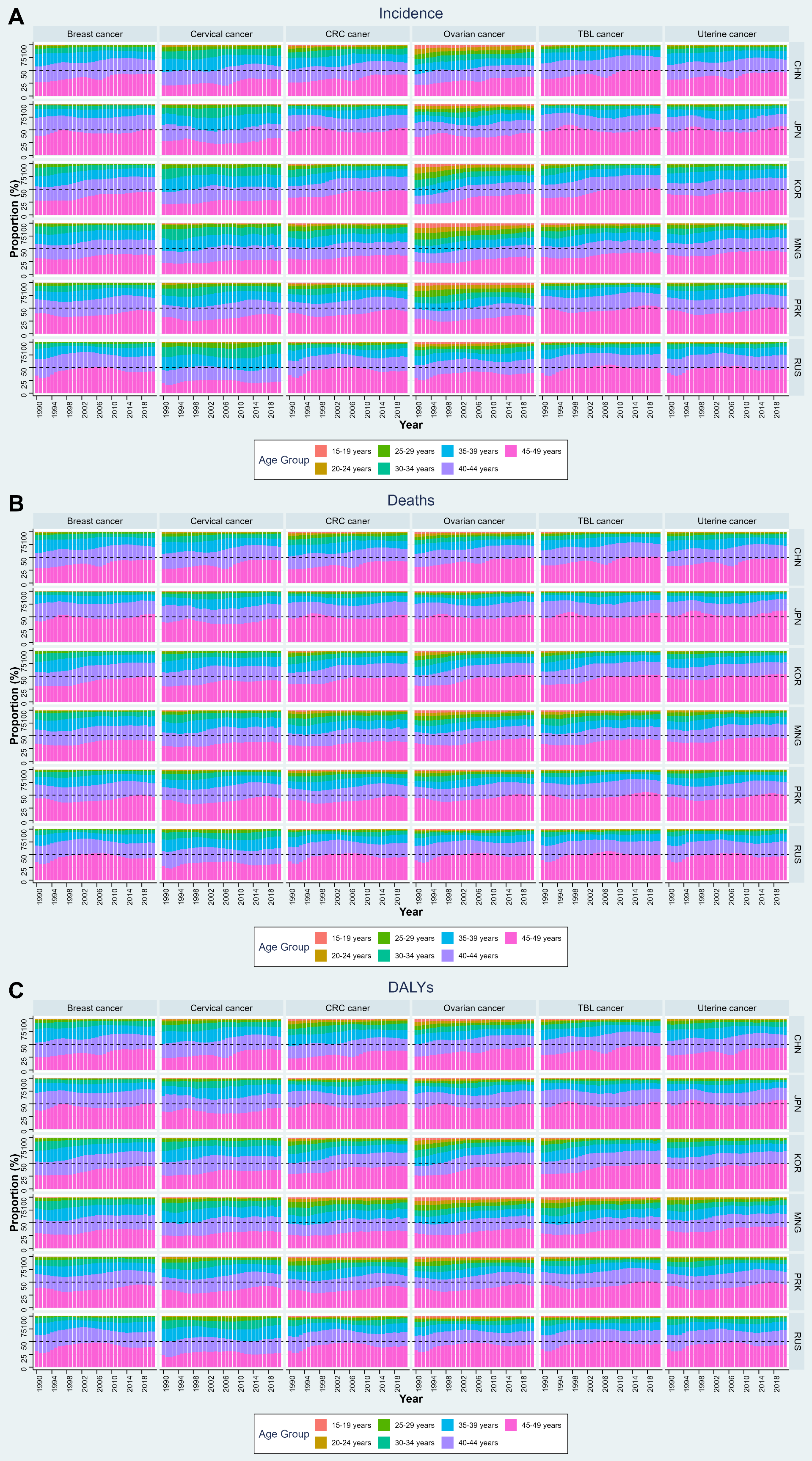


Figure S2. Age-specific composition of cancer burden among WCBA stratified by countries, 1990–2021. (A) Proportion of incidence, (B) proportion of deaths, and (C) proportion of DALYs, by 5-year age groups. Stacked bars indicate the relative contribution of each age subgroup (15–19, 20–24, 25–29, 30–34, 35–39, 40–44, and 45–49 years) to the total burden of each cancer type. NEA, Northeast Asia. WCBA, women of childbearing age (15-49 years). DALYs, disability-adjusted life years. TBL cancer, tracheal, bronchus, and lung cancer. CRC caner, colon and rectum cancer. CHN, China. JPN, Japan. KOR, Republic of Korea. MNG, Mongolia. PRK, Democratic People's Republic of Korea. RUS, Russian Federation.


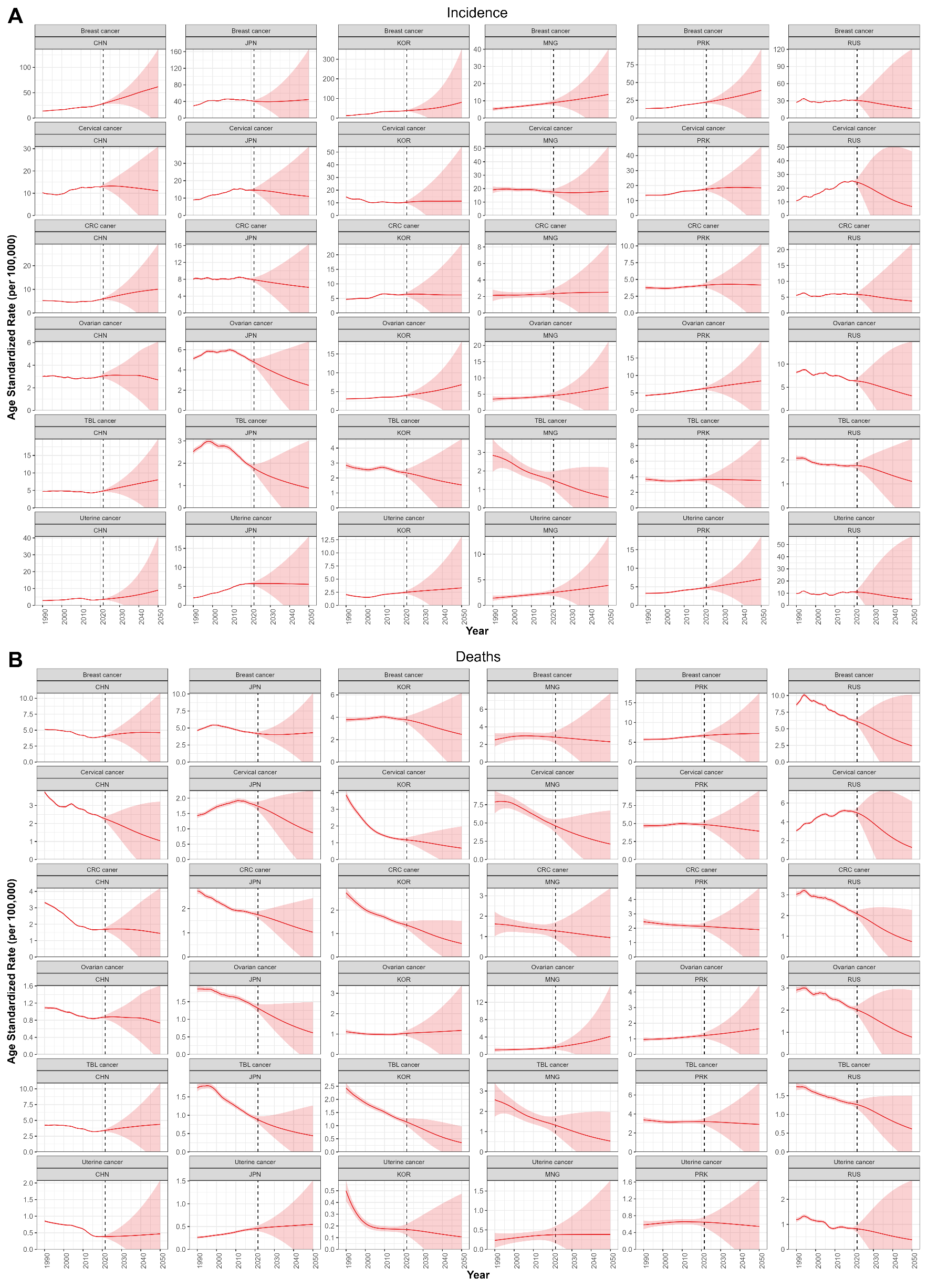


Figure S3. Projections of incidence (A) and mortality (B) rates for six selected cancers among WCBA stratified by countries through 2050 using the BAPC model. Solid red lines indicate observed and projected age-standardized rates (per 100,000), and shaded areas represent 95% uncertainty intervals. Vertical dashed lines mark the transition between observed data (1990–2021) and projections (2022–2050). NEA, Northeast Asia. WCBA, women of childbearing age (15-49 years). BAPC, Bayesian age–period–cohort. TBL cancer, tracheal, bronchus, and lung cancer. CRC caner, colon and rectum cancer. CHN, China. JPN, Japan. KOR, Republic of Korea. MNG, Mongolia. PRK, Democratic People's Republic of Korea. RUS, Russian Federation.
